# Evolutionary robotics simulations help explain why reciprocity is rare in nature

**DOI:** 10.1038/srep32785

**Published:** 2016-09-12

**Authors:** Jean-Baptiste André, Stefano Nolfi

**Affiliations:** 1 Institut des Sciences de l’Evolution, Université de Montpellier, CNRS, IRD, EPHE CC 065 Place Eugène Bataillon 34095 Montpellier cedex 05, France; 2 CNR, Institute of Cognitive Sciences and Technologies, Laboratory of Autonomous Robots and Artificial Life, Via S. Martino della Battaglia 44, 00185, Roma, Italy

## Abstract

The relative rarity of reciprocity in nature, contrary to theoretical predictions that it should be widespread, is currently one of the major puzzles in social evolution theory. Here we use evolutionary robotics to solve this puzzle. We show that models based on game theory are misleading because they neglect the mechanics of behavior. In a series of experiments with simulated robots controlled by artificial neural networks, we find that reciprocity does not evolve, and show that this results from a general constraint that likely also prevents it from evolving in the wild. Reciprocity can evolve if it requires very few mutations, as is usually assumed in evolutionary game theoretic models, but not if, more realistically, it requires the accumulation of many adaptive mutations.

Evolutionary models inspired by game theory have shown in detail how reciprocal cooperators can invade populations of selfish individuals^1^,^2^,^3^,^4^,^5^. The pros and cons of various strategies have been compared, including the famous “Tit-for-Tat”, which replicates the partner’s past actions^1^, “Generous Tit-for-Tat”, which tolerates one episode of defection^4^, or “Pavlov”, which changes behavior after receiving a low payoff 2,5. The overall conclusions of this line of research are that (i) reciprocal cooperation can emerge through evolution from a state of defection under the effect of demographic stochasticity and/or clustering^1^,^2^,^6^, and (ii) reciprocal cooperation can be evolutionarily stable provided that some form of phenotypic variability is maintained^7^,^8^,^9^,^10^,^11^,^12^,^13^.

Empirical observations are, however, at odds with these theoretical results^14^,^15^,^16^,^17^,^18^,^19^,^20^. Relatively few instances of reciprocity – strictly defined as the exchange of actions whose (potential) benefits depend on the responses of others – have been demonstrated in nature beyond the human case. Empirical researchers have argued that reciprocity is intrinsically difficult to demonstrate, and rightly pleaded for a broad definition that is not based on a literal interpretation of models and is therefore compatible with real life settings^21^. It has even been demonstrated that some interactions in the wild are in fact reciprocal^22^,^23^,^24^,^25^,^26^,^27^,^28^,^29^. However, these qualifications notwithstanding, reciprocal cooperation is a marginal mechanism outside the human case. Findings on cooperation in nonhuman species show that it is underpinned by nonreciprocal mechanisms in the overwhelming majority of cases. Kin selection and common interests, most importantly, have played a pivotal role in the history of life, allowing the emergence of new levels of organization such as genomes, multicellular organisms, and complex societies^30^,^31^. In comparison, the ecological impact of reciprocity is very moderate at most. The resulting puzzle is this: why, despite the apparent theoretical plausibility of reciprocity, is it so much rarer than other forms of cooperation?

In a recent theoretical paper, one of us proposed a simple hypothesis to solve this puzzle^32^, suggesting that reciprocity has rarely evolved because it raises an evolutionary problem of “bootstrapping” of the same kind as for the evolution of communication: it requires the joint evolution of several functions at the same time. Even though it may be adaptive once it has evolved (i.e., reciprocity can be an evolutionarily stable strategy) reciprocity cannot be shaped gradually by natural selection. Instead its evolution requires specific facilitating mechanisms, which explain both its relative rarity and the actual form that it takes in extant species^33^.

This hypothesis rests upon the assumption that the *mechanisms* underlying behavior are such that the emergence of a fully functional reciprocating phenotype by mutation, from an initial state of pure defection, is highly unlikely. In evolutionary game theory models^32^, however, mechanistic constraints, and the resulting mapping between genotype and phenotype, must be a priori assumed. The objective of the present paper is thus to test the hypothesis by following an alternative approach in which these constraints emerge naturally from the model itself. To do so, we used evolutionary robotics, a particularly useful methodology to study the evolution of social behavior^34^,^35^.

We simulated the evolution of a population of digital agents (thereafter called robots) controlled by artificial neural networks (see Methods). The main task of robots was to forage as a pair, assuming that they stayed with the same partner throughout the simulation, making it easy for them to reciprocate without needing to recognize their partner. To give the robots opportunities to help each other, we assumed that, with a constant probability per unit of time, each robot became stuck at a random position in the environment, preventing it from foraging. Once stuck, a robot could break free on its own, which we assumed to occur at a low rate, or it could be released five times faster if its partner moved close and stayed until its release – a helping behavior that entailed a cost in lost foraging time. Because we wished to separate the problem of the evolution of cooperation from the problem of the evolution of communication, we assumed that a robot’s color changed when it was stuck, making it easy for its partner to detect when it needed help.

We first considered robots that perceived their current environment but could not remember the past. In this first phase we simulated the evolution of a population of 100 robots under two conditions. In the *unrelated* condition, every robot was paired on each evaluation with a random partner sampled from the entire population excluding the focal robot itself (in this case, the partners’ genetic relatedness was *R* = 0). In the *related* condition, each robot was always paired with a clone of itself (the partners’ genetic relatedness was *R* = 1). In both conditions, we ran 10 independent simulations for 500 generations each, extensively measuring the “helping rate” of robots every 25 generations (see Methods for details).

In all 10 simulations, robots in genetically unrelated pairs evolved into efficient foragers with a very low helping rate. In contrast, the helping rate of robots with genetically related partners rose to high values (comparison of the related and unrelated conditions, Mann-Whitney U-test *p*-value < 0.001; Fig. 1a and movies S1 and S2). We then compared the helping rate of the best individual at the end of each simulation with the rate found when its partner was invisible (and hence helping could only occur by chance). As predicted, in the *unrelated* condition robots helped their partners at less than chance level – i.e., they actively avoided helping (collisions are often costly in robotics because it is difficult to separate from an immobile physical object following a collision; 0.00640234 ± 0.0102107 < 0.0424987 ± 0.00353933; Wilcoxon signed-rank test *p*-value < 0.01) – whereas in the *related* condition robots helped at more than chance level – i.e., they actively sought to help (0.150628 ± 0.0217893 > 0.0386472 ± 0.00475379; Wilcoxon signed-rank test *p*-value < 0.01).

Behavioral observations showed that robots that evolved in this condition would typically forage until they detected a stuck partner, at which point they stopped foraging and concentrated on releasing their partner (movie S2). Because robots in genetically related pairs mutually benefited from each other’s help, their average payoff was higher (Fig. 1b). In summary, helping entails a social benefit but an individual cost and, therefore, it evolves only if partners are genetically related^36^.

We then considered robots with the ability to retain some information on their partner’s past behavior. To do so, we gave the robots an additional input neuron, called the “memory” neuron, whose activation reflected the ratio of the number of times the individual had received help in the past to the total number of times it had been stuck (see Methods). The robots thus always had access to information regarding their partner’s past propensity to help. The question was whether evolution would favor robots that use this information to behave reciprocally. We ran 10 independent simulations with genetically unrelated pairs of robots for 2000 generations. In each simulation, robots evolved the ability to forage efficiently (not shown), but (i) their helping rate remained very low (Fig. 1 and supplemental information [SI], Fig. SI1), not significantly greater than that of memoryless robots (Mann-Whitney U-test *p*-value ≈ 0.15), and (ii) the rate at which they had been helped in the past did not affect how much they helped their partner (see Fig. SI1) – i.e., reciprocity did not evolve.

It could be that this negative result was an idiosyncratic consequence of the specific nature of our experiment. Two possibilities in particular must be considered. First, the neural controller of our robots might have been unable to express reciprocal helping at all. Second, reciprocal cooperation could simply have happened not to be adaptive in the scenarios we examined, for instance because interactions were not sustained for a long enough period. In either case, the non-evolution of reciprocity would be an artifactual consequence of our specific assumptions.

To rule out these two explanations, we bred reciprocal helpers by artificial selection. First, we evolved a population of robots while “paying” them (i.e., increasing their fitness) for helping when their memory neuron was highly activated (reflecting the fact that one’s partner has been helpful in the past), while punishing them for helping in the opposite case (see Methods for details). After 1500 generations of this selection regime, we obtained a very efficient conditional helper, which we called Reciprocator, which helped only when its memory neuron was highly activated (see movies S3 and S4, and see SI, Fig. SI2). This rules out the first possibility: the neural controller was in fact able to express conditional helping.

To rule out the second possibility – that reciprocity was simply not adaptive in our setting – we created two other genotypes by artificial selection (see SI): a Selfish genotype that forages efficiently and almost never helps (movie S5), and a Helper genotype that also forages efficiently but that always helps a partner in need (movie S6). We measured the payoffs of individuals with these two genotypes, as well as Reciprocators, when partnered with individuals of each of the three types (Table 1). The resulting payoffs were in line with standard game theory. In particular, Reciprocators were not favoured when very rare among Selfish partners, but could invade when their frequency rose above an invasion barrier (because they can benefit from interactions with other reciprocators^4^).

Using these payoffs, we were then able to simulate the evolutionary dynamics of the three genotypes under the assumption of a mutational distance of 1 between each pair of genotypes (see SI). Also in line with standard game theory^37^, this yielded cycles of cooperation and defection in small populations (Fig. 2a,b), and a stable polymorphism with cooperation predominating in large populations (Fig. 2c). This shows that reciprocity can evolve, and even predominate, if it is only a single mutation away from defection. To further evaluate the potential role of reciprocity in evolving populations, we then performed another set of experiments in which we let populations of robots evolve while introducing the Reciprocator genotype artificially at a constant rate per generation (1 individual per generation on average; see SI for details). In each of the 15 simulations run in this condition, reciprocity was maintained and led to relatively large levels of helping at equilibrium (Fig. 1 and Fig. SI3).

Reciprocal helpers can thus be bred by artificial selection, and they are able to invade populations of selfish individuals. And yet reciprocity never arises spontaneously during evolution. To understand what constraints prevent this emergence, we examined the composition of our 10 evolved populations (Fig. 3). The vast majority of individuals in these populations were plain defectors. A small fraction of individuals, however, sometimes helped their partner, while a still smaller fraction helped at a rate that depended on the activation of their memory neuron, which could be a stepping stone toward the evolution of reciprocity. Interestingly, however, all conditional helpers had relatively low fitness, and in fact were only present at mutation-selection balance owing to the relatively high mutation rate used in our simulations (see SI). Behavioral observations showed that some were so attracted by their partner that their foraging rate was reduced – they would “forget to eat” (movie S7) – whereas others collided with their partner and were then unable to separate (movies S8 and S9), and still others helped inefficiently (movie S10). This did not occur in the well-optimized Reciprocator, who helped efficiently when needed, and kept on foraging otherwise (movie S3). But reaching such a fine-tuned balance between attention to one’s partner and motivation to forage requires the accumulation of many adaptive mutations. The first random mutations generating conditional helping carry significant fitness costs.

To confirm this analysis, in a further experiment we artificially selected for efficient conditional helping while also favoring similarity to the reference Selfish genotype (see SI). The 62 reciprocating genotypes obtained in this way differed from the Selfish genotype at an average of 25 different loci (positions in the neural network), and the closest genotype differed at 15 loci – i.e., it took at least 15 independent mutations to transform a plain defector into an efficient reciprocal helper.

These results were obtained in a particular evolutionary robotic setting. They do not constitute a general proof that reciprocity can never evolve in biology – and in fact this is not true, since reciprocity has evolved in some cases. They nevertheless bring to light a general principle explaining why the evolution of reciprocity is unlikely except under specific biological conditions (see below), supporting our original hypothesis^32^. The first mutations that confer a novel biological function are always imperfect. But normally the function is improved later on by further mutations owing to the action of a directional selective pressure. The problem with reciprocity is that it is favored only if a significant fraction of other individuals in the population already reciprocate. Hence, until full-fledged reciprocity is present, no directional selective pressure can cause such gradual improvement, and the first random mutants with imperfect reciprocal abilities cannot be refined by further evolution. Reciprocity can evolve only if it appears immediately in fully-fledged form, by the occurrence of just the right mutations (see SI for a stylized model capturing this idea). Such mutants occur in game-theoretic models in which one or a very small number of random mutations suffice to transform a plain defector into an efficient reciprocator. But they are very unlikely to occur in a realistic setting in which reciprocity is an actual behavior and must be produced by a cognitive system.

Game-theoretic models are misleading because they neglect the fact that behaviors must be generated by actual *mechanisms*. When the selective pressure acting on behavior depends on the behavior of other individuals, then evolution is path-dependent, and the mechanistic underpinnings of behavior have qualitative consequences. In the case in point, the fact that reciprocity is a complex behavioral adaptation in mechanistic terms makes its evolution highly unlikely, in contrast to theoretical predictions.

This result leads to the opposite puzzle, however. Reciprocal cooperation does exist in a number of non-human species^22^,^23^,^24^,^25^,^26^,^27^,^28^,^29^, and there is no doubt of its existence in humans. Hence, reciprocity must sometimes be able to evolve in spite of the bootstrapping problem identified above. Using our simple paradigm, we tested two simple scenarios that could facilitate its evolution. First, we wanted to know whether the pre-evolution of an unconditional form of helping (e.g., due to genetic relatedness) could also facilitate the evolution of conditional helping. To investigate this question, we initialized 10 independent populations with the artificial Helper genotype and let them evolve for 1000 generations in the absence of genetic relatedness. This did not lead to the evolution of conditional helping (see Fig. SI4). Instead, selection always led to a rapid reduction of helping, before any conditionality could arise. Second, we wanted to test Axelrod and Hamilton’s hypothesis that reciprocity could evolve as a by-product of kin recognition when the degree of relatedness varies from partner to partner, the idea being that helping itself then plays the role of a kin recognition criterion^1^. To do so, we initialized 10 populations randomly and let them evolve for 1000 generations with an intermediate level of genetic relatedness (individuals interacted with a clone of themselves with probability 1/2 and a random partner with complementary probability), and then 1000 generations in the absence of relatedness. Reciprocity also failed to evolve in this scenario (see Fig. SI5). Helping was not a relevant trait on which to base kin recognition because it did not remain polymorphic (a monomorphic trait, by definition, cannot be used to recognize who is kin and who is not^38^). Clearly, these two simple scenarios did not generate reciprocity for the same reason as the standard scenario: because they lacked a directional selective pressure in favor of a *conditional* form of cooperation.

In a recent model, one of us proposed that the evolution of reciprocity would be more likely in another type of scenario^33^. Namely, reciprocity can emerge when: (i) a small amount of cooperation initially evolves as a by-product of a self-serving trait (or kin selection), and (ii) the return on benefit of this cooperation partly depends upon others’ cooperation. In this case, it is genuinely beneficial for individuals to act conditionally, plastically adapting their cooperative investment to that of others. This is the essence of reciprocity, and can lead to a quantitative expansion of cooperation beyond what is initially favored for selfish reasons. This process can occur, for instance, when cooperation has synergistic benefits, such as in collective actions, or in symmetrical interactions: that is, in situations in which help in one direction makes it easier or more beneficial for others to help in the other direction^33^. Testing this type of scenario is, however, beyond the scope of the present paper, and should be the object of further evolutionary robotics simulations.

## Methods

### Experimental setup

Robots’ behavior was controlled by a three-layer feed-forward neural network. The input layer contained 1 neuron with a constant activation of unity, and 17 sensory neurons, including 8 proximity sensors distributed uniformly around the robot, and 9 camera neurons detecting red, green, and blue colors in a 60° cone (3 neurons per color, 20° per neuron, the activation of each camera neuron being proportional to the portion of the visual field that was filled with the corresponding color). Input neurons’ activation varied in the range [0.0, 1.0]. The hidden layer contained 9 neurons. The output layer consisted of 2 neurons controlling the velocity of the robot’s wheels, which could vary in the range [−8.2, 8.2] cm/s. Both hidden and motor neurons had a sigmoid activation function. The state of the sensors, motors, and environment was updated every 100 ms. The 182 connection weights of the neural network, which determined the robot’s behavior, were encoded in an artificial genotype.

The environment contained two cylindrical robots with a diameter of 7 cm and two immobile food sources with a diameter of 5.4 cm located in a 1 *m* × 1 *m* toroidal area. Robots gained 1 fitness unit each time they moved over (“ate”) a food source, in which case the food source disappeared and was immediately replaced by another food source at a random position in the arena. Robots were normally mobile and green, but they became stuck with constant probability of 5 · 10^−4^ per unit of time, which caused their color to turn blue. Stuck robots broke free (and turned green again) naturally at a rate of 5 · 10^−4^ per unit of time, or 5 times faster if their partner came sufficiently close (less than 1 cm) and remained there (“helping”). Each evaluation of a pair of robots lasted for 20.000 time steps. Each robot was evaluated 5 times with different partners (*unrelated* condition), or 5 times with a clone of itself (*related* condition). An individual’s fecundity was defined as the number of food items it gathered during these 5 evaluations.

Populations initially consisted of robots with random connection weights. At each generation, once all robots had been evaluated, the next generation was randomly sampled in proportion to individual fecundity, according to a Wright-Fisher process. The connection weights were subjected to mutations during reproduction (each connection weight was encoded as a binary number with 8 digits, and mutations occurred at each digit with probability of 10^−2^).

### Behavioral assays

Each individual was placed 100 independent times at a random position in an environment with two food sources and one stuck partner (which, we assumed, could not release itself) and tracked for 1000 time steps. We measured (i) the proportion of times the individual released its partner and (ii) the average time it took to do so. Using these two measures, by maximum likelihood, we estimated each individual’s probability of helping per time step, which we call the helping rate.

### Memory neuron

Each occasion when a robot was stuck constituted an opportunity for it to receive help, which could either culminate in an actual helping event (the robot’s partner releases it) or not (the robot becomes free without help). The memory neuron measured the number of helping events out of the total number of opportunities to receive help in the robot’s past, which was between 0 and 1. The neuron was set at its maximum value of 1 at the beginning of each evaluation.

### Artificial selection in favor of reciprocity

At the beginning of each evaluation, individuals were assigned a random value ∈ {0, 1} which was artificially fed to their memory neuron and kept constant throughout the evaluation. If their memory neuron was set to 1, their fecundity increased if they helped their partner, and if their memory neuron was set to 0, helping decreased their fecundity. We ran 4 independent simulations with 200 individuals each for 1500 generations (using a steady-state genetic algorithm: see SI). We obtained 800 genotypes in total, many of which cooperated conditionally (not shown). To select among these genotypes, we then calculated their probability of fixation, under a Moran process, if introduced as a single copy in a population of 100 individuals where the other 99 played the reference Selfish strategy (see SI), and we chose the genotype with the maximum fixation probability.

## Additional Information

**How to cite this article**: André, J.-B. and Nolfi, S. Evolutionary robotics simulations help explain why reciprocity is rare in nature. *Sci. Rep.* **6**, 32785; doi: 10.1038/srep32785 (2016).

## Supplementary Material

Supplementary Movie S1

Supplementary Movie S2

Supplementary Movie S3

Supplementary Movie S4

Supplementary Movie S5

Supplementary Movie S6

Supplementary Movie S7

Supplementary Movie S8

Supplementary Movie S9

Supplementary Movie S10

Supplementary Information

## Figures and Tables

**Figure 1:**
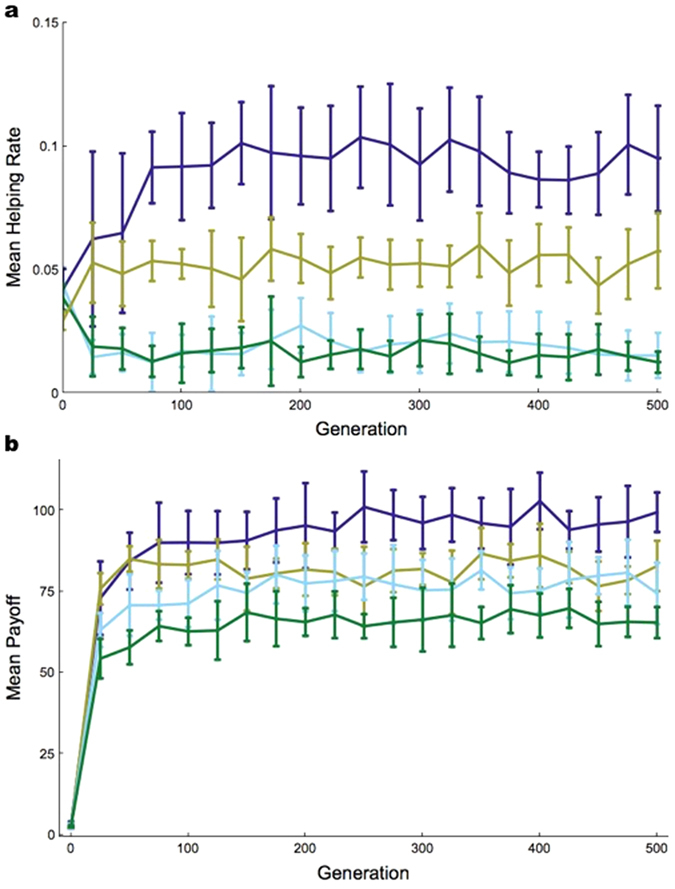
Evolution of helping and average payoff through time. Here we show the evolution of helping rates (**a**) and average payoffs (**b**) in evolutionary robotics simulations, averaged across 10 independent simulation runs, in 4 different conditions. Error bars indicate the standard deviation obtained across runs. Dark and light blue curves show the outcome of simulations performed in the absence of a memory neuron, in the *related* and *unrelated* conditions respectively. Robots helped more and obtained a larger average payoff in the *related* condition. In green, we show the results obtained in robots with a memory neuron (but in the absence of genetic relatedness), for the first 500 generations of evolution (see Fig. SI1 for the remaining 1500 generations). Remembering their partner’s past actions did not lead robots to help more at the evolutionary equilibrium (compare the green and light blue curves in (**a**)). Having a memory neuron even led to a reduction in the average payoff (green curve in (**b**)), probably because it introduced more perturbations into the network. The brown curves shows the average result of 5 simulations when the artificial genotype “Reciprocator” was introduced by hand during simulations (with a probability of 10^−2^ per individual per generation). The helping rate and the average payoff in this case were intermediates (see Fig. SI3 for further results where the “Reciprocator” was introduced by hand).

**Figure 2:**
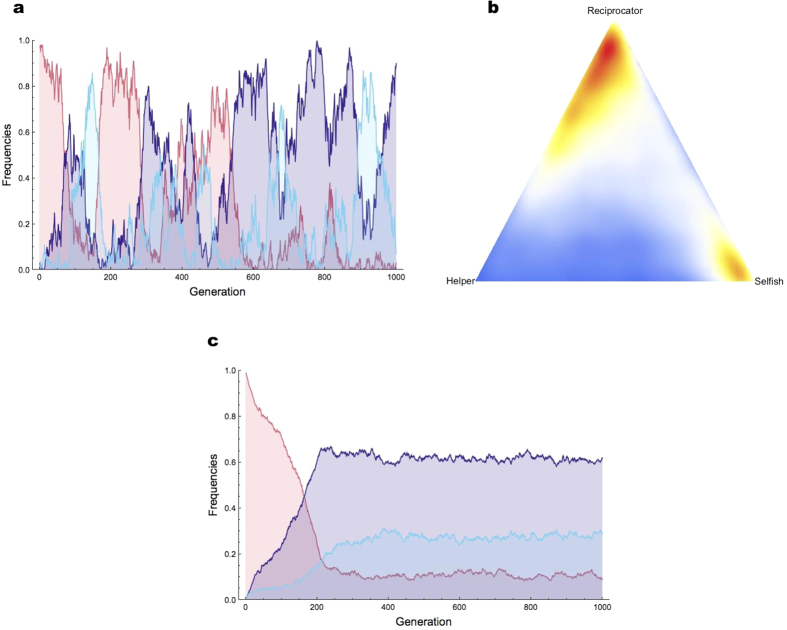
Evolutionary dynamics of Selfish, Reciprocator, and Helper. Here we simulate the evolutionary dynamics of the three genotypes from their payoffs as shown in Table 1 in a population of 100 individuals, or 10^4^ individuals. In (**a**,**c**) we plot the evolution of genotype frequencies through time in the small population and large population case, respectively (Selfish is red, Reciprocator is dark blue, and Helper is light blue). In (**b**), we show the state-space representation of 50.000 generations of evolution in the small population case. The number of visits to a point in the state-space is indicated by its color (red points correspond to states that are often visited; blue points to states that are rarely visited). In the small population case, evolutionary dynamics oscillated between a state with a majority of Reciprocators mixed with some Helpers, and a state with a majority of Selfish individuals. In the large population case, the population reached a stable polymorphism with a majority of Reciprocators.

**Figure 3:**
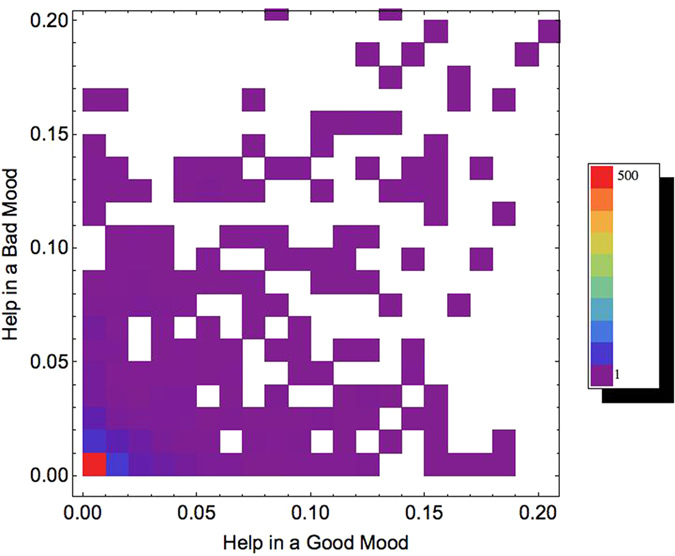
Composition of evolved populations. We show the composition of the 10 independent populations of robots (endowed with a memory neuron) after 2000 generations of evolution. The two axes respectively represent the helping rate of robots when their memory neuron is activated at its maximum level (called “help in a good mood”) or at its minimum level (called “help in a bad mood”). The color code indicates the number of robots who expressed the corresponding helping rates among the 10 evolved populations (all populations pooled together). The vast majority of individuals expressed low helping rates in both conditions.

**Table 1:** Payoffs of the row player in all possible encounters, measured by simulating 1000 interactions per pair.

	*Selfish*	*Helper*	*Reciprocator*
*Selfish*	152	205	160
*Helper*	132	198	175
*Reciprocator*	147	203	173

This shows that the interaction between Selfish and Helper is a prisoner’s dilemma in which selfishness is evolutionarily stable, whereas helping is mutually preferable. When Reciprocator is introduced into a population of Selfish players, Reciprocator becomes favored by selection iff its frequency rises above a threshold frequency, which is equal to 0.27 with the above payoffs.
