# Supplementary material for: Evolutionary robotics simulations help explain why reciprocity is rare in nature
Source: Sci Rep. 2016 Sep 12;6:32785. doi: 10.1038/srep32785 (PMC5018820; doi:10.1038/srep32785)
Supplement: Supplementary Information [file srep32785-s11.pdf]

Supplemental Information for J.B. André and S. Nolfi

"Evolutionary robotics simulations help explain why reciprocity is rare in nature"

May 2, 2016

Steady-state genetic algorithm

We considered a population of 200 individuals. Every generation, (i) each individual was evaluated (here, 5 times), and (ii) each individual generated one random mutant which was also evaluated. Each mutant's fitness was then compared to the lowest fitness in the parent population. If the mutant was better, it replaced the incumbent. This algorithm allowed us to rapidly explore a large genotypic space. When used with genetically unrelated pairs it allowed us to breed the reference Selfish genotype, and when used with genetically related pairs it allowed us to breed the reference Helper genotype.

Fixation probability in a Moran process

As a proxy for the potential success of a given reciprocating genotype, we calculated its fixation probability in a Moran process, when a single copy was introduced in a population of competitors playing a reference selfish strategy. Call P_{ss} the payoff of a selfish individual when interacting with another selfish individual, P_{sr} the payoff of a selfish individual when interacting with a reciprocating individual, P_{rs} the payoff of a reciprocating individual when interacting with a selfish individual, and P_{rr} the payoff of a reciprocating individual when interacting with another reciprocating individual. In the Moran process, the exact fixation probability of a single reciprocator introduced in a population of $N-1$ selfish individuals is given by Karlin

and Taylor (1975) [1] as:

$$F_{rs} = \left(1 + \sum_{k=1}^{N-1} \prod_{n=1}^{k} \frac{P_s(n)}{P_r(n)}\right)^{-1} \quad (1)$$

where $P_s(n) = \frac{n}{N-1}P_{sr} + \frac{N-n-1}{N-1}P_{ss}$ is the average payoff of a selfish individual in a population containing n reciprocating individuals and $N-n$ selfish individuals, and $P_r(n) = \frac{n-1}{N-1}P_{rr} + \frac{N-n}{N-1}P_{rs}$ is the average payoff of a reciprocating individual in a population containing n reciprocating individuals and $N-n$ selfish. This exact expression was calculated numerically for every reciprocating strategy using the software Mathematica.

Evolution when Reciprocators are introduced artificially

To evaluate the potential role of reciprocity in natural populations, we ran evolutionary robotic simulations in which Reciprocators were introduced artificially. In each generation, in addition to normal random mutations, every individual could mutate toward the Reciprocator genotype with probability 10^{-2}. This means that in a population of 100 individuals, an average of one Reciprocator was introduced per generation. We ran 5 simulations in this condition for 1000 generations. The results are shown in Fig. 1 of the main text (purple curve), and in Fig. SI3a. The average levels of helping and conditionality obtained were intermediate, in between those obtained with genetically unrelated pairs and those obtained with genetically related pairs (orange and green curves in Fig. 1 of main text). As in our experiment with

regular evolution, these simulations were initialized with random genotypes. We also ran 5 complementary versions of the same experiment where we initialized each population with the artificial Selfish genotype (previously obtained by optimization; see main text). This yielded similar results (Fig. SI3b). Finally, we also performed the same experiment 5 times while introducing the three genotypes (Reciprocator, Selfish and Helper) at the same rate, yielding again similar results (Fig. SI3c).

Simulating the evolutionary dynamics of the three genotypes

We consider a finite population of constant size N, evolving under a Wright-Fisher process. We assume that only three genotypes can exist: Selfish, Helper, and Reciprocator, with payoffs as given by Table 1 of main text. The population initially contains only Selfish individuals. Simulations follow the number of individuals of each genotype across generations under the effect of mutation, selection, and drift. The fitness of each genotype in a given generation is given by the sum of its payoffs against all genotypes, weighted by their respective frequency (i.e., we assume that each individual interacts with a representative sample of the population). The composition of the population in the next generation is obtained by sampling N individuals among the offspring of the previous generation, in proportion to their fitness. At reproduction,

each individual can mutate with probability 10^{-2} toward one of the two other genotypes (e.g. a Selfish individual has a probability 5.10^{-3} of becoming a Reciprocator, and a probability 5.10^{-3} of becoming a Helper, etc.).

Detailed examination of the composition of natural populations

We measured the fitness of each genotype in every population (by comparing each individual to all the other genotypes in its population). Due to the high mutation rate, fitness was highly variable in our populations – i.e., each population contained many deleterious genotypes with relatively low fitness at mutation-selection balance. Fig. 3 of the main text shows the composition of our 10 independently evolved populations after 2000 generations. In Fig. SI6 we plot the composition of the subset of individuals in the same populations whose fitness was at least 95% of the maximum in the population. In this case, all the conditional helpers disappear, which shows that conditional helping was always associated to deleterious genotypes, because the mutations that led to conditional helping also imposed significant fitness costs.

Selection for similarity to a reference Selfish genotype

In order to estimate the minimum number of mutations needed to transform a pure defector into an efficient reciprocal helper, we artificially selected for conditional cooperation using a steady-state genetic algorithm (see SI) in the following way: (i) We initialized the population with all individuals playing the reference Selfish strategy. (ii) We specifically introduced mutations toward the Selfish genotype: random mutation at each locus led to the Selfish genotype at that locus with a probability of 1/2. (iii) We directly selected for similarity to the Selfish genotype lexicographically: when the fitness of two genotypes differed by more than 5%, their degree of similarity to the Selfish genotype was disregarded, but when two genotypes had similar payoffs (within 5%), we then favored the one whose genotype was most similar to the Selfish genotype. We performed 5 independent artificial selection experiments in this way, with 200 individuals each, for 1000 generations, leading to a total of 1000 possible reciprocators.

For each possible reciprocator, we then measured P_{ss}, P_{sr}, P_{rs}, and P_{rr}, the payoffs obtained in all possible combinations of encounters with Selfish individuals, and calculated from equation (1) the fixation probability F_{rs} of a single individual with this genotype introduced into a population of 99 Selfish competitors evolving under a Moran process. Among the 1000 candidate reciprocators, we then kept only the genotypes that satisfied the following three conditions: (i) $P_{rr} > P_{ss}$, i.e., two reciprocators did better when partnered with each other than did two Selfish

individuals; (ii) $P_{rr} > P_{sr}$, i.e., a reciprocator did better than a Selfish individual when paired with another reciprocator, and (iii) $F_{rs} > 0.01$, i.e., the reciprocator's fixation probability was above neutral. In this way, we obtained 62 reciprocators with the potential to play an important role in evolution. Among these 62 genotypes, the average number of differences with the Selfish reference genotype was 25, the maximum was 36, and the minimum was 15. Hence, it took at least 15 independent mutations to transform the Selfish genotype into an efficient reciprocator.

Stylized model

We considered a fixed population with a selfish strategy: that is, a genotype that produced individuals who were unable to help and unable to detect or respond to others' helping. We examined the effect of a random mutation improving the reciprocating ability of this genotype and sought to derive the fixation probability of this mutation. We assumed that the random mutation led individuals to cooperate with efficiency ϵ and with conditionality ρ (see below).

The social payoff obtained by a selfish individual partnered with another selfish individual was $P_{ss} = 0$, since they did not cooperate. The social payoff of a reciprocator mutant paired with another reciprocator was $P_{rr} = b - c(\epsilon)$, where b is the benefit of being helped and $c(\epsilon) = b \times (1 - \epsilon)$ is the cost of helping, which decreased with the mutant's efficiency ϵ. The social payoff of a selfish individual paired with a reciprocator was $b \times (1 - \rho)$, which decreased

with the degree of conditionality ρ of the reciprocator. Finally, the social payoff of a reciprocator paired with a selfish individual was $-c(\epsilon) \times (1 - \rho)$, because the reciprocator would cooperate with probability $(1 - \rho)$ despite its partner's systematic defection.

The average social payoff of a selfish individual in a population containing n reciprocators and $N - n$ selfish individuals was $P_s(n) = \frac{n}{N-1} P_{sr} + \frac{N-n-1}{N-1} P_{ss}$. The average social payoff of a reciprocator in a population containing n reciprocators and $N - n$ selfish individuals was $P_r(n) = \frac{n}{N-1} P_{rr} + \frac{N-n-1}{N-1} P_{rs}$. An individual's fecundity was a function of its average social payoff. However, in order to avoid negative fecundities, we also assumed that individuals obtained a fraction $1 - \sigma$ of their fecundity from sources other than the focal social interaction. Hence, the fecundity of an individual of type i in a population including n reciprocators was given by $W_i(n) = \sigma P_i(n) + 1 - \sigma$, where $i \in \{s, r\}$.

The Moran fixation probability of a single reciprocator introduced in a population of $N - 1$ selfish individuals can be calculated like in equation (1) as:

$$F_{rs} = \left(1 + \sum_{k=1}^{N-1} \prod_{n=1}^{k} \frac{W_s(n)}{W_r(n)}\right)^{-1} \quad (2)$$

which is then numerically evaluated and plotted as a function of ϵ and ρ (Fig. SI7). This shows that, in order to have a non-negligible probability of fixation, a reciprocator mutant must express a form of helping that is *both* highly efficient and highly conditional, which is unlikely to occur by random mutation.

References

[1] S Karlin and H. M Taylor. *A First Course in Stochastic Processes, Second Edition*. Academic Press, 1975.

Figure captions

Figure SI1. Evolution with a memory neuron Here we show the mean helping rate (blue) and degree of behavioral conditionality (yellow) averaged across 10 independent evolutionary simulations with robots endowed with a memory neuron (same simulations as the blue curves of Fig. 1 of main text). The degree of conditionality is measured as the difference between the helping rate when the memory neuron was activated at its minimum (0) versus its maximum level (1). The helping rate of the best individual in the last generation of each run is independent of the activation of its memory neuron (Wilcoxon signed-rank test p-value ≈ 0.65).

Figure SI2. Behavior of Reciprocator The helping rate of the artificially bred Reciprocator for various levels of activation of its memory neuron when the partner was either visible (red bars) or invisible (and helping thus occurred by chance: gray bars). Reciprocator helped its partner more than chance when its memory neuron was activated, but less than chance when memory neuron was not activated.

Figure SI3. Evolution when the Reciprocator is introduced artificially In (**a**), we show the mean helping rate (blue) and degree of conditionality (yellow) averaged across 5 independent evolutionary simulations with robots endowed with a memory neuron, when the

Reciprocator was introduced by hand with a probability of 10^{-2} per individual per generation (same simulations as the purple curve of Fig. 1 of main text). In (**b**), we show the average result of 5 simulations performed in the same condition except that we initialized each population with the artificial Selfish genotype. In (**c**), we show the average result of 5 further simulations in which we introduced each of the three artificial genotypes (Selfish, Reciprocator, and Helper) with the same probability of 10^{-2} per individual per generation.

Figure SI4. Evolution initialized with the Helper genotype Here we show the mean helping rate (blue) and degree of conditionality (yellow) averaged across 10 simulations performed in the same condition as in Fig. SI1, except that simulations were initialized with the reference Helper genotype. Selection led to a rapid reduction of the average Helping, before conditionality could evolve.

Figure SI5. Evolution with intermediate relatedness Here we show the mean helping rate (blue) and degree of conditionality (yellow) averaged across 10 simulations performed in the same condition as in Fig. SI1, except that genetic relatedness was $R = 0.5$ for the first 1000 generations (individuals interacted with a clone of themselves with probability 1/2 and a random partner with complementary probability), and then $R = 0$ for the next 1000 generations. Selection led to an intermediate level of helping in the first 1000 generations, and then to a reduction of helping in the following 1000 generations. Conditional cooperation was

never favored.

Figure SI6. Composition of evolved populations We show the composition of the 10 independent populations of robots (endowed with a memory neuron) after 2000 generations of evolution, as in Fig. 3 of main text, except that here only individuals with at least 95% of the maximum fitness are included. The two axes represent the helping rate of robots when their memory neuron was maximally activated (called “help in a good mood”) or minimally activated (called “help in a bad mood”). The color code indicates the number of robots who expressed the corresponding helping rates among the 10 evolved populations (all populations pooled together). All individuals with high fitness expressed low helping rates in both conditions.

Figure SI7. Selection for reciprocity in the stylized model Fixation probability of random mutants, multiplied by population size, in the stylized model described in SI. Any mutant with a value above 1 is better than neutral, and vice versa. The benefit of helping was $b = 5$, the population size was 100, and the strength of selection was $\sigma = 0.1$.

Movie captions

In all movies, we artificially increased the rate at which robots became stuck and released (as compared to evolutionary simulations) in order to observe more helping events. All movies except movie S4 show interactions between two robots with the same genotype.

Movie S1. A memoryless robot obtained after 500 generations of evolution in the *unrelated* condition.

Movie S2. A memoryless robot obtained after 500 generations of evolution in the *related* condition.

Movie S3. A conditional helper, called Reciprocator, obtained by artificial selection.

Movie S4. Interaction between a Reciprocator and a Selfish robot (the Selfish robot is stuck at the beginning of the movie).

Movie S5. The reference Selfish genotype.

Movie S6. The reference Helper genotype.

Movie S7. A conditional helper present at mutation-selection balance after 2000 generations of evolution. Individuals with this genotype are so attracted by their partner that their foraging rate is reduced.

Movie S8. A conditional helper present at mutation-selection balance after 2000 generations of evolution. Individuals with this genotype tend to collide with their partner and are then unable to separate.

Movie S9. A conditional helper present at mutation-selection balance after 2000 generations of evolution. Individuals with this genotype tend to collide with their partner and are then unable to separate.

Movie S10. A conditional helper present at mutation-selection balance after 2000 generations of evolution. Individuals with this genotype help in an inefficient manner.

Figure S11

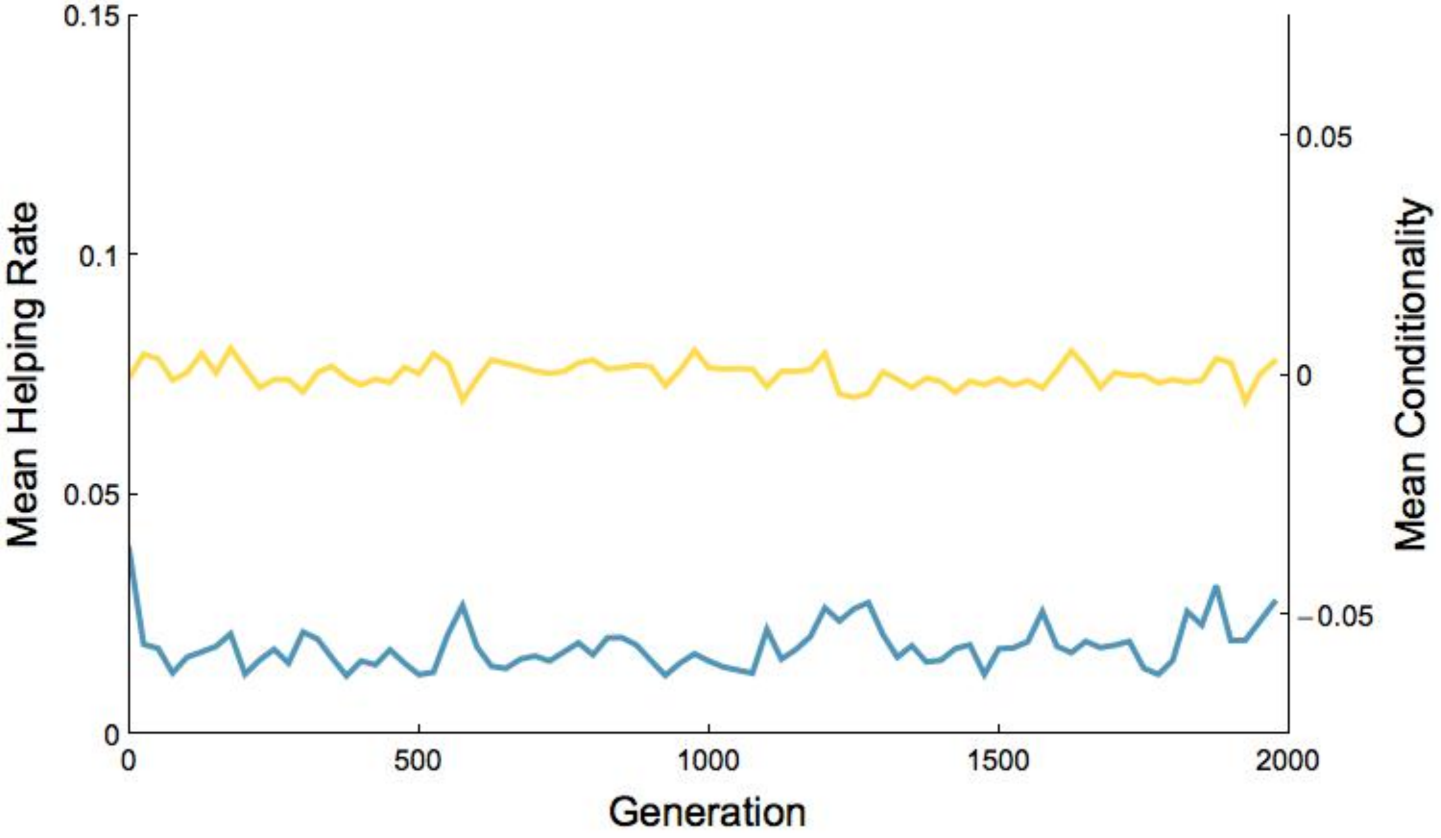
0.15
0.1
0.05
0
Mean Helping Rate
0.05
0
−0.05
Mean Conditionality
0
500
1000
1500
2000
Generation

Figure SI2

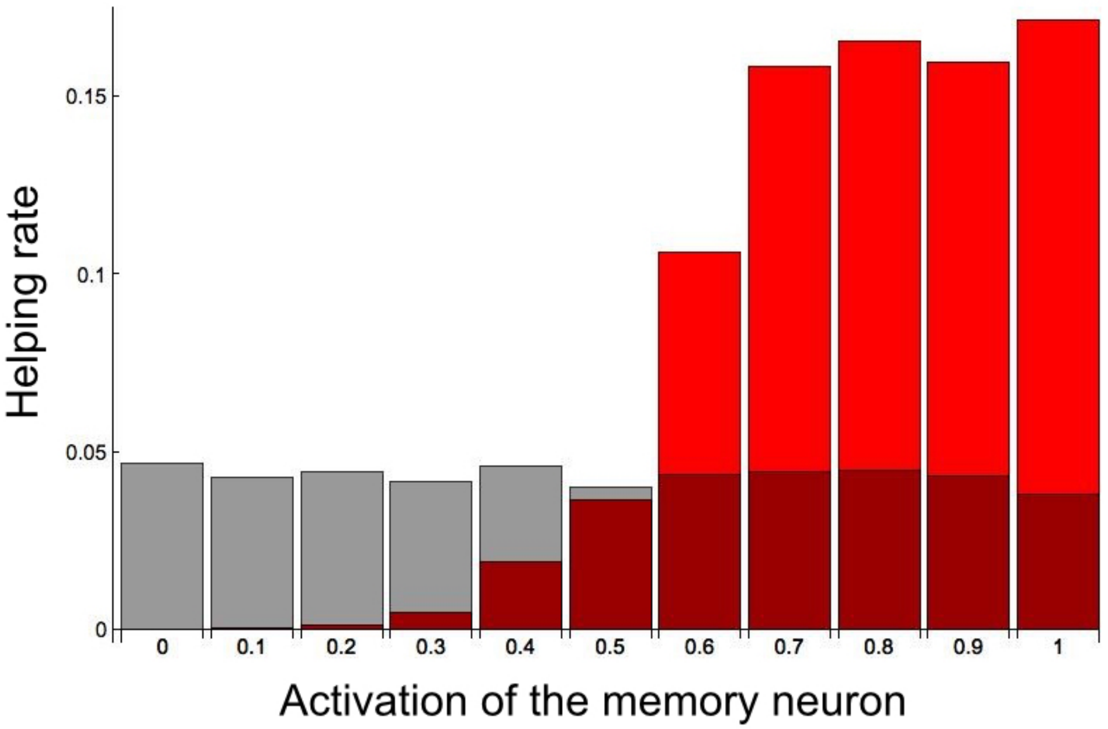

Helping rate
0.15
0.1
0.05
0
0
0.1
0.2
0.3
0.4
0.5
0.6
0.7
0.8
0.9
1
Activation of the memory neuron

Figure SI3

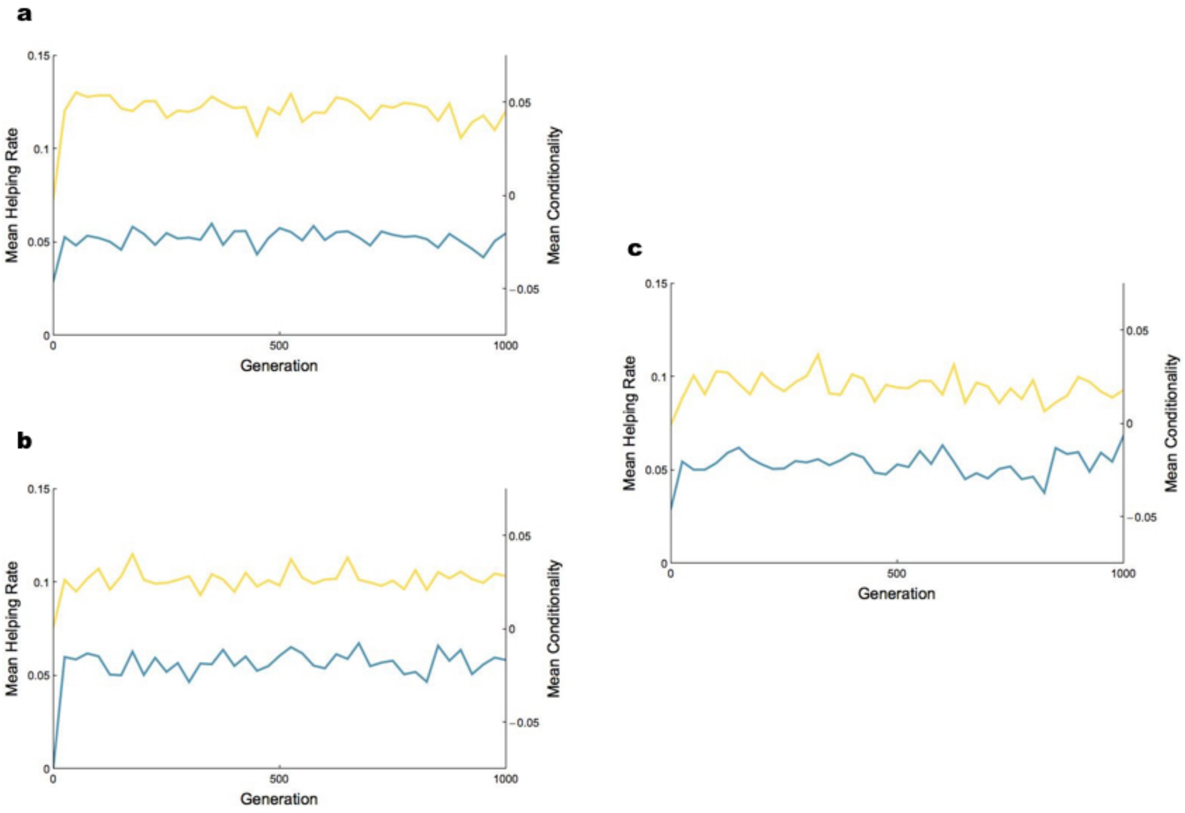
a
0.15
0.1
0.05
0
Mean Helping Rate
0.05
0
−0.05
Mean Conditionality
0
500
1000
Generation
b
0.15
0.1
0.05
0
Mean Helping Rate
0.05
0
−0.05
Mean Conditionality
0
500
1000
Generation
c
0.15
0.1
0.05
0
Mean Helping Rate
0.05
0
−0.05
Mean Conditionality
0
500
1000
Generation

Figure SI4

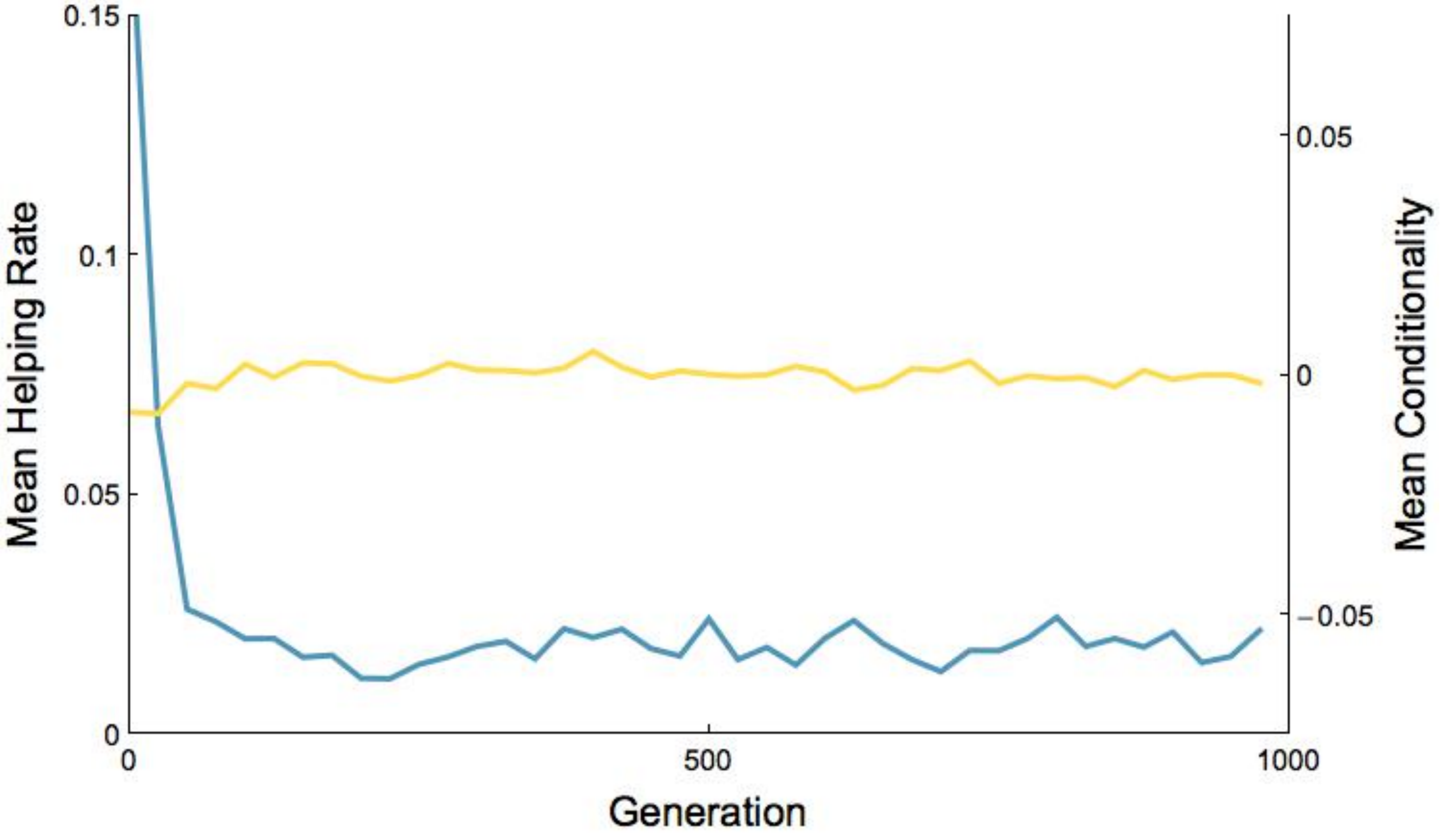
Mean Helping Rate
Mean Conditionality
Generation
0.15
0.1
0.05
0
0.05
0
−0.05
0
500
1000

Figure SI5

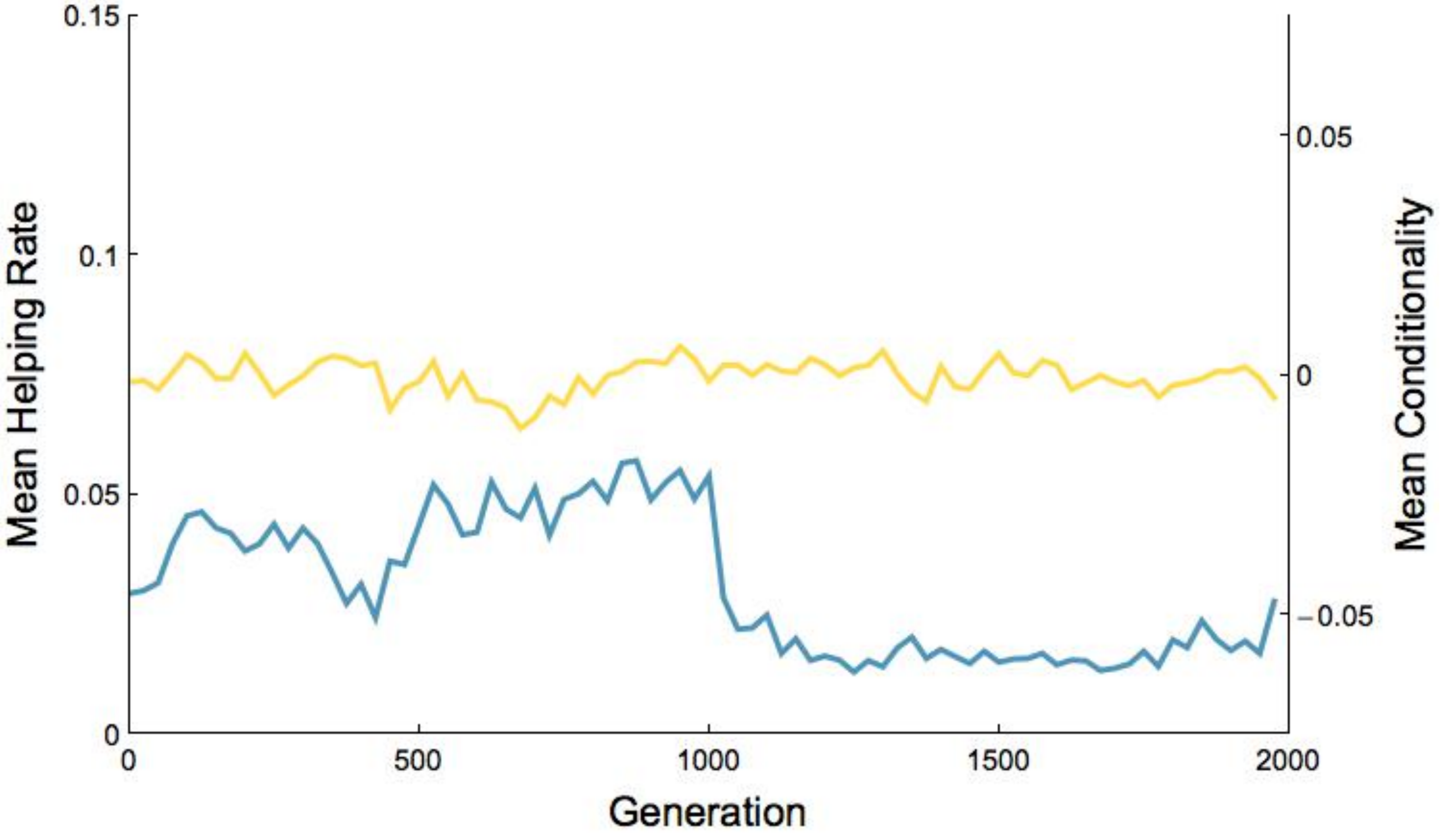

0.15
0.1
0.05
0
Mean Helping Rate
0.05
0
−0.05
Mean Conditionality
0
500
1000
1500
2000
Generation

Figure SI6

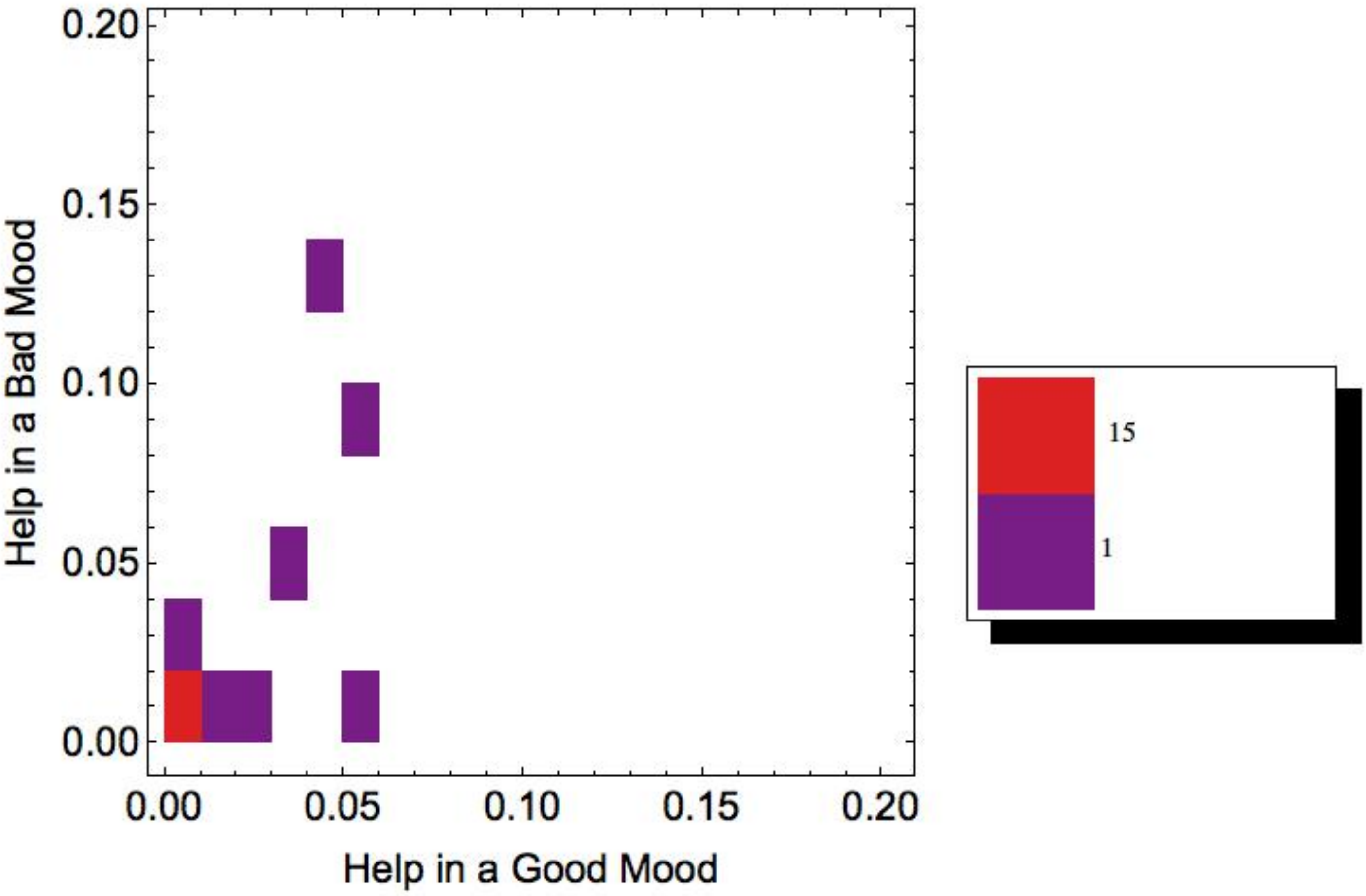
0.20
0.15
0.10
0.05
0.00
Help in a Bad Mood
0.00
0.05
0.10
0.15
0.20
Help in a Good Mood
15
1

Figure SI7

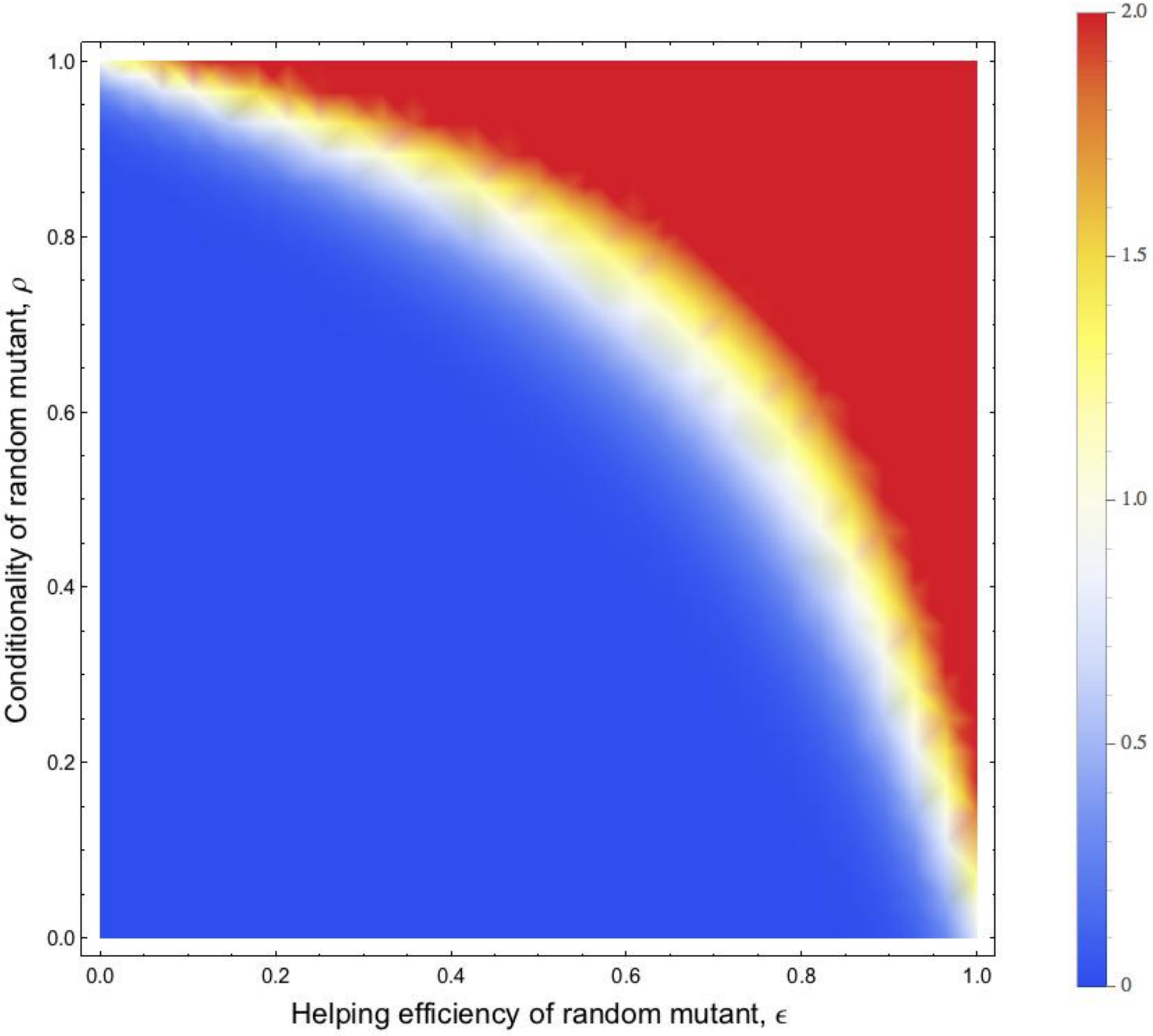

Conditionality of random mutant, ρ
Helping efficiency of random mutant, ϵ
0.0
0.2
0.4
0.6
0.8
1.0
0
0.5
1.0
1.5
2.0
